# O-GlcNAc modifications regulate cell survival and epiboly during zebrafish development

**DOI:** 10.1186/1471-213X-9-28

**Published:** 2009-04-21

**Authors:** Danielle M Webster, Chin Fen Teo, Yuhua Sun, Dorota Wloga, Steven Gay, Kimberly D Klonowski, Lance Wells, Scott T Dougan

**Affiliations:** 1 Department of Cellular Biology, The University of Georgia, Athens, GA 30602, USA; 2 Complex Carbohydrate Research Center, Department of Biochemistry and Molecular Biology, The University of Georgia, Athens, GA 30602, USA; 3 Current address: Hodges University, School of Allied Health, Naples, FL 34119, USA

## Abstract

**Background:**

The post-translational addition of the monosaccharide O-linked β-*N*-acetylglucosamine (O-GlcNAc) regulates the activity of a wide variety of nuclear and cytoplasmic proteins. The enzymes O-GlcNAc Transferase (Ogt) and O-GlcNAcase (Oga) catalyze, respectively, the attachment and removal of O-GlcNAc to target proteins. In adult mice, Ogt and Oga attenuate the response to insulin by modifying several components of the signal transduction pathway. Complete loss of *ogt *function, however, is lethal to mouse embryonic stem cells, suggesting that the enzyme has additional, unstudied roles in development. We have utilized zebrafish as a model to determine role of O-GlcNAc modifications in development. Zebrafish has two *ogt *genes, encoding six different enzymatic isoforms that are expressed maternally and zygotically.

**Results:**

We manipulated O-GlcNAc levels in zebrafish embryos by overexpressing zebrafish *ogt*, human *oga *or by injecting morpholinos against *ogt *transcripts. Each of these treatments results in embryos with shortened body axes and reduced brains at 24 hpf. The embryos had 23% fewer cells than controls, and displayed increased rates of cell death as early as the mid-gastrula stages. An extensive marker analysis indicates that derivatives of three germ layers are reduced to variable extents, and the embryos are severely disorganized after gastrulation. Overexpression of Ogt and Oga delayed epiboly and caused a severe disorganization of the microtubule and actin based cytoskeleton in the extra-embryonic yolk syncytial layer (YSL). The cytoskeletal defects resemble those previously reported for embryos lacking function of the Pou5f1/Oct4 transcription factor *spiel ohne grenzen*. Consistent with this, Pou5f1/Oct4 is modified by O-GlcNAc in human embryonic stem cells.

**Conclusion:**

We conclude that O-GlcNAc modifications control the activity of proteins that regulate apoptosis and epiboly movements, but do not seem to regulate germ layer specification. O-GlcNAc modifies the transcription factor Spiel ohne grenzen/Pou5f1 and may regulate its activity.

## Background

The activities of key proteins must be controlled in a precise spatio-temporal manner for proper embryonic development. This is accomplished by regulation at the transcriptional, translational and post-translational levels. While the effects of post-translational modifications such as phosphorylation, proteolytic cleavage and N-linked glycosylation have been well studied, relatively little is known about how the O-linked addition of the simple monosaccharide β-*N*-acetylglucosamine (O-GlcNAc) affects protein activities during development. This sugar is attached to a wide variety of nuclear and cytoplasmic proteins, including those implicated in controlling important steps during development such as NeuroD1, β-catenin, c-myc and the cytoplasmic tails of plakoglobin and E-cadherin [1-5]. Other proteins modified by O-GlcNAc include casein kinase II (CKII), nuclear pore proteins, RNA polymerase II and numerous transcription factors [6-15]. These observations implicate O-GlcNAc in the regulation of transcription, protein stability and cell cycle progression, among other events [7,8,16,17]. Attachment of O-GlcNAc is rapidly induced in response to external stimuli such as nutrient status and stress, and is reversible [18,19]. Thus, unlike complex glycosylation, O-GlcNAc modification is highly dynamic and transiently alters protein function in a manner analogous to phosphorylation [20].

O-GlcNAc is covalently linked to serine and threonine residues of target proteins by the enzyme O-GlcNAc Transferase (Ogt), which uses the product of the hexosamine biosynthetic pathway, UDP-GlcNAc, as a high-energy substrate [21,22]. The reverse reaction is catalyzed by O-GlcNAcase (Oga), a cytosolic and nuclear N-acetylglucosaminidase [23,24]. Both Ogt and Oga are modified by O-GlcNAc. The residues of many proteins modified by O-GlcNAc are also targets of ser/thr kinases, suggesting that Ogt modulates signal transduction pathways by preventing phosphorylation kinase targets [20,25]. This model of regulatory control by O-GlcNAc modification is supported by studies of the roles of Ogt and Oga in the insulin pathway. Insulin shuts down glucose production in hepatocytes by promoting the phosphorylation of the CRTC2 transcription factor, which is then targeted for ubiquitin-dependent degradation [26]. By O-GlcNAcylating CRTC2, Ogt prevents its phosphorylation and protects it from degradation [27]. CRTC2 then antagonizes the effects of insulin by inducing expression of gluconeogenic genes such as glucose-6-phosphatase [28]. Ogt also antagonizes the response to insulin in adipocytes by modifying and inactivating a number of components of the insulin signal transduction cascade, including IRSs and PI3K [29,30]. When overexpressed in adult mice, Ogt induces insulin resistance whereas Oga suppresses the development of type II diabetes [27,29,31].

In humans, a single *ogt *gene produces three enzymatic isoforms [32]. Two isoforms are localized to the nuclei and cytoplasm, while one isoform appears to be localized exclusively to the mitochondria [33]. Ogt contains two conserved catalytic domains in the C-terminus, called CDI and CDII, and an N-terminal tetratricopeptide repeat domain (TPR) that differs in the three isoforms and mediates protein-protein interactions [34,35]. Although the monomer can catalyze the addition of O-GlcNAc in *in vitro *assays, multimeric Ogt has a greater affinity for UDP-GlcNAc [34]. This suggests that Ogt forms an active multimeric complex *in vivo*. In support of this idea, the TPR domain is essential for enzyme activity in *Xenopus *oocytes [36]. The recent solving of a bacterial Ogt homolog has shed light on the activity and specificity of the enzyme [37].

The role of O-GlcNAc modification in regulating embryonic development has been more difficult to establish. In mice, *ogt *is expressed in embryonic stem cells and in all adult tissues examined [38]. The complete absence of *ogt *function is lethal to mouse embryonic stem cells and embryos, but the phenotype has not been well characterized [38]. Experiments with conditional alleles revealed that *ogt *is required in a tissue specific manner at later stages [39]. Differentiated neurons survive in the absence of *ogt *function, but the brains are reduced and animals survive less than 10 days. By contrast, embryonic fibroblasts do not proliferate in the absence of *ogt *function, and mutant T-cells undergo apoptosis. In *Xenopus *oocytes, O-GlcNAc levels fluctuate during oogenesis and remain elevated after fertilization until the onset of gastrulation [36]. Ogt activity is required for oocyte maturation and blocking its activity prevents entry into M-phase [36,40]. These results show that *ogt *is required for cell proliferation at early stages, but has stage and tissue dependent functions at later stages. We were interested in determining the function of *ogt *during the blastula and gastrula stages, when the body axes are established and the germ layers form.

*ogt *is highly conserved throughout evolution, and orthologues are found in at least three eukaryotic kingdoms, including animalia, plantae, and fungi [41,42]. Genetic studies in the plant *Arabidopsis thaliana *show that it is possible to identify new roles for *ogt *by analyzing hypomorphic mutant conditions. There are two genes in *Arabidopsis*, called *spindly *(*spy*) and *secret agent *(*sec*) [42]. *spy; sec *double mutant seeds fail to germinate, indicating that the two genes have overlapping requirements in early development [43]. Thus, the requirement of *ogt *for proliferation in early embryos has been strongly conserved in plants and animals. In *spy *single mutants, SEC provides sufficient Ogt activity to bypass the early requirement, revealing a later requirement. *spy *mutant plants display an aberrant response to the plant growth hormone, gibberellin [44,45]. Therefore in plants, Ogt acts at later stages of development to regulate specific signalling pathways, analogous its role modulating the response to insulin in mammals.

To determine if O-GlcNAc modification modulates signalling pathways that control vertebrate development, we used the zebrafish model organism. There are two *ogt *orthologues in zebrafish, present in tandem copies on chromosome 14, separated by approximately 200 kb [46]. The two genes are highly similar at the nucleic acid level, and are expressed maternally and zygotically. The genes have two alternatively spliced exons in zebrafish *ogt*, producing six different enzyme isoforms [46]. We show that both full-length and short Ogt isoforms are active *in vitro *and *in vivo*, in contrast to previous reports [46]. To determine the role of O-GlcNAc modifications in development, we increased or decreased O-GlcNAc levels in embryos by overexpressing zebrafish Ogt, human Oga, or morpholinos against *ogt *transcripts. Each treatment results in smaller embryos with increased rates of cell death. In addition, Ogt and Oga overexpressing embryos have morphogenesis defects, probably resulting from underlying defects in the actin and microtubule based cytoskeleton in the yolk. Finally, we show that the pou class transcription factor Oct4/Pou5f1 is modified by O-GlcNAc in human embryonic stem cells. This suggests that O-GlcNAc regulates the activity of Pou5f1 in embryonic stem cells and, possibly, in whole embryos. We conclude that O-GlcNAc modifications play a critical role during embryogenesis in regulating morphogenesis and cell survival.

## Results

### Two *ogt *genes arose from a zebrafish specific gene duplication

**Figure 1:**
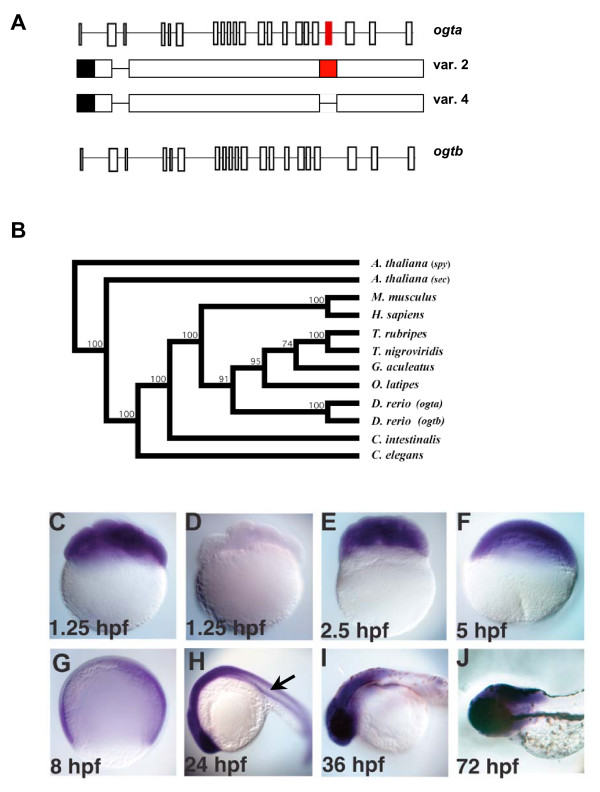
**The origin and expression pattern of zebrafish *ogt *genes**. (A) Schematic diagram of zebrafish *ogta *and *ogtb *loci (formerly known as copy I and copy II, respectively), which are located on Chromosome 14 [46]. Diagrams of variant 2 and 4 transcripts from the *ogta *gene are represented under the diagram of the *ogta *locus. The black box represents the 3' UTR, while the red boxes in the *ogta *locus and variant 2 transcript represent the alternatively spliced exon 19, which has been proposed to inhibit O-GlcNAc Transferase activity [46]. Exon 19 is absent from variant 4 transcripts. (B) Phylogenetic analysis of *ogt *genes constructed using the neighbor-joining method using aligned amino acid sequences (see materials and methods for gene identifier numbers for each of the sequences used). The *A. thaliana ogt *gene *spy *was used to root the tree, since it is the most divergent *ogt *gene we analyzed. Bootstrap values for 1,000 repetitions are indicated at the nodes. (C-J) A time course of *ogt *gene expression from 8-cell (1.25 hpf) (C) to 72 hpf (J) of development reveals that *ogt *is dynamically expressed in early embryos. *ogt *is ubiquitously expressed in cleavage stage (C, E), blastula stage (F) and gastrula stage (G) embryos. Signal is not detected in embryos incubated with sense probe (D). At 24 hpf, *ogt *transcripts are expressed at high levels in the brain and lower levels trunk and tail (H). At 36 hpf (I) and 72 hpf (J), *ogt *transcripts are restricted to the brain.

Unlike other animals, zebrafish have two *ogt *genes, called copy I and copy II, which are 67% identical at the nucleotide level and 89% identical at the amino acid level [46]. To conform to the standard zebrafish nomenclature, we rename these genes *ogta *and *ogtb*, respectively (Fig. 1A). Alternative splicing of *ogta *produces four transcripts (variants 1–4), while alternative splicing of *ogtb *produces two transcripts (variants 5 and 6) [46]. The plant *Arabidopsis thaliana *is the only other model organism with two *ogt *genes, *spindly *(*spy*) and *secret agent *(*sec*) [42]. We performed a phylogenetic analysis to determine when the duplication event occurred. *Danio rerio ogta *is 37% identical to *sec *at the amino acid level and only 21% identical to *spy*. Similarly, *Danio rerio ogtb *is 39% identical to *sec*, and only 22% identical to *spy*. Thus, both zebrafish genes are more closely related to one another and to *sec *than to *spy*. This indicates that *Danio rerio ogta *and *ogtb *originated after the plant and animal kingdoms diverged (Fig. 1B). To pinpoint more precisely when the *ogt *duplication event occurred, we examined the *ogt *genes in the sequenced genomes of other metazoan animals, including worms, sea squirts, mice and humans [32,38,47,48]. Each of these species has a single *ogt *gene, raising the possibility that the zebrafish *ogt *gene duplication occurred during the teleost specific whole genome duplication event (Fig. 1B) [49]. To test this, we examined the sequenced genomes of other teleost fish for *ogt *orthologues. The green spotted pufferfish (*Tetraodon nigroviridis*), fugu pufferfish (*Takifugu rubripes*), stickleback (*Gasterosteaus aculeatus*) and medaka (*Oryzias latipes*) each have only a single *ogt *orthologue (Fig. 1B) [50-52]. We conclude that the *D. rerio ogta *and *ogtb *arose from a very recent, zebrafish specific gene duplication.

To investigate the function of *ogt *in zebrafish, we isolated a full-length *ogt *transcript from a 15–19 hours post fertilization (hpf) cDNA library. Sequence analysis revealed that this transcript encodes the variant 2 Ogt isoform, which lacks exon 2a and contains exon 19 (Fig. 1A, red boxes) [46]. Since previous experiments showed that bacterially expressed variant 2 Ogt lacked enzymatic activity [46], we removed exon 19 from this construct in order to generate an enzyme with the same sequence as the variant 4 isoform (Fig. 1A) [46].

### *ogt *expression is gradually restricted to the brain

A previous study included an extensive RT-PCR analysis of expression of all Ogt variants over a developmental time course [46]. We examined the spatial distribution of *ogt *transcripts by *in situ *hybridization in a developmental time-course (Fig. 1C–J). Our probe was complementary to the entire variant 2 sequence and is expected to hybridize to all transcripts from the *ogta *and *ogtb *genes due to the high homology at the nucleotide level between the two genes. *ogt *transcripts are found in all cells of cleavage stage embryos, consistent with earlier reports of maternal expression (Fig. 1C) [46]. This staining was specific, since no signal was detected with a sense probe (Fig. 1D). High levels of *ogt *transcripts persist in all cells through the blastula stages (Fig. 1E, F), but decline during gastrulation (Fig. 1G). At 24 hpf, the staining is more intense in neural tissues, especially the brain, than in mesodermal tissues like the notochord (Fig. 1H, arrow). From 36 hpf to 72 hpf, *ogt *transcripts are localized exclusively in the brain (Fig. 1I, J). This demonstrates that *ogt *has a more dynamic expression pattern than previously suspected, consistent with the idea that it has different functions at different stages of embryonic development.

### O-GlcNAc modifications regulate embryonic development

**Figure 2:**
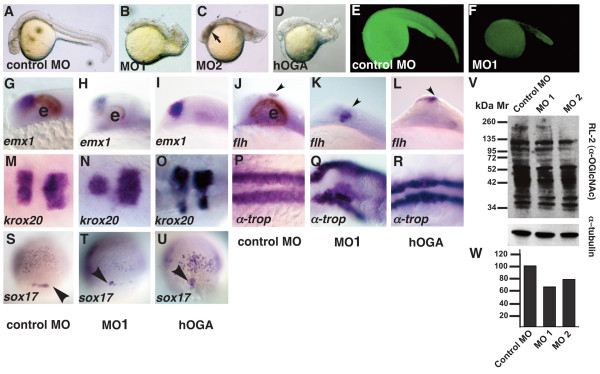
**Tissues from all three germ layers are reduced in *ogt *morphants**. Comparison of *ogt *morphants and hOga expressing embryos with controls at 24 hpf (A-R) or 8 hpf (S-U). (B) Embryos injected with 7.5 ng MO1 (B), MO2 (C) or hOga (D) were smaller than controls (A), and had curved tails, small brains, reduced or absent eyes and shortened body axes. Necrotic tissue in the head from the MO injection is indicated (C, arrow)[86]. An Ogt-gfp fusion protein is translated in the presence of control MOs (E), but not in the presence of MO1 (F). In embryos injected with MO1 (H, K, arrowhead) or hOga (I, L, arrowhead), *emx1 *expression is slightly reduced compared to controls (G) and *flh *expression in the epiphysis is normal or slightly expanded. *krox20 *is expression is reduced in *ogt *morphants (N) and hOga expressing embryos (O). α-*tropomyosin *is reduced and disorganized in *ogt *morphants (Q) and hOga overexpressing embryos (R), reflecting the disorganization of the entire body axis. Fewer *sox17 *expressing endodermal precursor cells and dorsal forerunner cells are apparent in *ogt *morphants (T, arrowhead) and hOga overexpressing embryos (U, arrowhead). (V) Extracts from embryos injected with control MO (lane 1), MO1 (lane 2) or MO2 (lane 3) were probed with the RL2 monoclonal antibody (upper) or anti-tubulin antibody (lower). (W) Embryos injected with the control MO have 35% more O-GlcNAc modified protein than embryos injected with MO1 and 20% more than embryos injected with MO2. Anterior is to the left in A-R, dorsal views in S-U. e = eyes. Representative embryos are shown.

**Figure 3:**
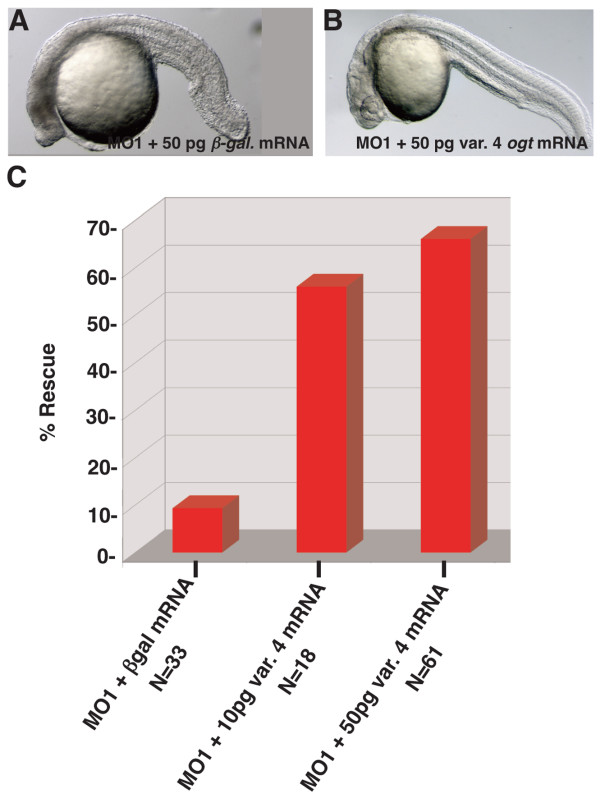
**Rescue of *ogt *morphants by injection of *variant 4 ogt *mRNA**. Lateral views of live *ogt *morphant injected with 50 pg β-*galactosidase *mRNA (A) or 50 pg *ogta variant 4 *mRNA (B). The image in A depicts the mild range of defects observed in morphants. (C) Graph depicting the frequency of wild type embryos in *ogt *morphants injected with 50 pg β-*galactosidase *mRNA (lane 1), 10 pg *ogta variant 4 *mRNA (lane 2), or 50 pg *variant 4 ogt *mRNA (lane 3). Injection of *ogta variant 4 *mRNA significantly increases the frequency of normal embryos.

Next, we took a loss-of-function approach to determine the function of *ogt *during embryonic development. We designed two translation-blocking morpholino oligonucleotides (MOs) against *ogt *transcripts. MO1 was designed to target all transcript variants of *ogta*, and MO2 was designed to target all transcript variants of *ogtb*. Translation blocking MOs inhibit the synthesis of protein from zygotic and maternal transcripts, but do not affect Ogt protein synthesized before the MO is injected. We reasoned that the early expressed protein might provide sufficient Ogt activity to bypass any requirement during the cleavage stages, permitting us to examine the role of Ogt at later stages of development. At 24 hpf, embryos injected with 7.5 ng of a control MO developed normally, and the eyes, midbrain-hindbrain boundary, notochord and somites are clearly visible (Fig. 2A; N = 40). By contrast, more than a third of embryos injected with 7.5 ng MO1 (34%) or MO2 (37%) were smaller than controls, with shortened body axes, reduced heads, small or absent eyes, and twisted notochords (Fig. 2B, C; N = 32, N = 35, respectively). Some embryos showed signs of necrosis in the head (Fig. 2C, arrow), which became more pronounced and more frequent with age (data not shown). When MO injected embryos are subsequently injected with *ogt *variant 4 mRNA, the fraction of wild type embryos increases from less than 10% to more than 50% (Fig. 3). This rescue rules out potential off-target effects of the MOs.

To confirm that the MOs reduce Ogt levels, we asked if they could efficiently prevent translation of an Ogt-gfp fusion protein. Embryos expressing *ogt-gfp *mRNA produce high levels of the fusion protein, as indicated by GFP fluorescence. Control MOs have no effect on fluorescence levels in these embryos (Fig. 2E; N = 30). In the presence of MO1, however, fluorescence levels decreased below the level of background yolk autofluorescence (Fig. 2F; N = 30). This suggests that the MO efficiently blocks translation of *ogt *transcripts and reduces the level of endogenous Ogt protein. To determine if the MOs reduced global O-GlcNAc levels, we performed Western blots on protein extracts from 18 hpf embryos using an O-GlcNAc specific antibody (Fig. 2V). For this analysis, we made protein extracts from embryos injected with MO1 or MO2 that displayed aberrant morphology and from embryos injected with a control MO that had normal morphology. We used the RL2 monoclonal antibody to recognize O-GlcNAc modified proteins (Fig. 2V). Quantification of all the bands revealed that total O-GlcNAc levels are reduced by 35% in embryos injected with MO1 as compared with controls, and by 20% in embryos injected with MO2 (Fig. 2V, W). This is consistent with a reduction in endogenous Ogt protein levels in MO injected embryos.

To determine if the defects in *ogt *morphants are due to reductions in O-GlcNAc levels, we expressed 250 pg mRNA encoding the human orthologue of *oga *(hOga; gi: 10835355). We found that hOga expressing embryos have a shortened body axis, reduced heads, small or absent eyes and twisted notochords (Fig. 2D). These defects strongly resemble those in *ogt *morphants, confirming that reducing O-GlcNAc levels has severe consequences on embryonic development. We conclude that O-GlcNAc modifications control the activity of proteins involved in controlling embryonic development.

### Derivatives of ectoderm, mesoderm and endoderm are reduced in *ogt*-depleted embryos

To understand the processes controlled by O-GlcNAc modifications, we performed an extensive analysis of the cell types present in *ogt *morphants by *in situ *hybridization. A table of the probes used and the tissues they mark is presented in the Methods (Table 1). We first examined expression of genes that mark derivatives of the neurectoderm, mesoderm or endoderm. *emx1 *marks the dorsal telencephalon in embryos injected with the control MO (Fig. 2G) [53]. The *emx1 *expression domain is slightly reduced in embryos injected with MO1 (Fig. 2H; N = 20) or MO2 (data not shown; N = 25). Another structure in the dorsal telencephalon, called the epiphysis, expresses the *floating head *(*flh*) transcription factor (Fig. 2J, arrowhead) [54]. Even in cases of dramatically reduced brains, *flh *is expressed normally in the forebrains of *ogt *morphants, or is expanded slightly (Fig. 2K, N = 30; MO2 data not shown, N = 30). The consistent expression of *emx1 *and *flh *indicates that the dorsal telencephalon forms and is normally patterned in *ogt *morphants. In the segmented hindbrain, rhombomeres 3 and 5 express the zinc finger transcription factor *krox20 *(Fig. 2M) [55]. In Ogt-depleted embryos, *krox20 *is still expressed in two stripes, although expression in rhombomere 3 is often reduced (Fig. 2N, N = 30; MO2 data not shown, N = 27). In embryos overexpressing hOga, *emx1 *is expressed in the dorsal telencephalon, as in *ogt *morphants (Fig. 2I; 4/6), and *flh *is expressed in the epiphysis (Fig. 2L; 5/7). In addition, *krox20 *transcripts are detected in rhombomeres 3 and 5, although rhombomere 3 is often reduced (Fig. 2O; 7/10). Thus, the anterior-posterior patterning of the brain is normal when O-GlcNAc levels are decreased, but the amount of tissue is slightly reduced in the forebrain and hindbrain.

Derivatives of the mesoderm and endoderm are reduced and disorganized when *ogt *function is reduced. Within the mesoderm, slow muscle forms in the somites on either side of the midline, and expresses α-*tropomyosin *(Fig. 2P) [56]. In the morphants, α-*tropomyosin *expression reveals the presence of slow muscle tissue in a reduced number of highly disorganized somites (Fig. 2Q, 12/19; MO2 data not shown 9/15). Endoderm precursors are apparent during gastrulation, as individual *sox17 *expressing cells migrate toward the animal pole (Fig. 2S) [57]. In addition, *sox17 *is expressed in a small group of dorsal forerunner cells (Fig. 2S, arrowhead), which are not of endodermal origin [58]. Endodermal expression of *sox17 *is significantly reduced in the morphants, indicating that the embryos have fewer endoderm precursors than embryos injected with control MOs (compare Fig. 2S and 2T). The morphants also have fewer dorsal forerunner cells (Fig. 2T). Embryos expressing hOga strongly resemble the morphants, and have fewer somites than controls (Fig. 2R; 5/7). In addition, hOga expressing embryos have significantly fewer endoderm precursors (Fig. 2U; 17/25) and dorsal forerunner cells than controls (Fig. 2U, arrowhead). The reduction of ectodermal, mesodermal and endodermal tissues in *ogt *morphants and hOga expressing embryos suggests that O-GlcNAc modifications regulate cell proliferation, survival, or cell fate determination.

### Induction of mesoderm and endoderm are normal in *ogt *morphants

**Figure 4:**
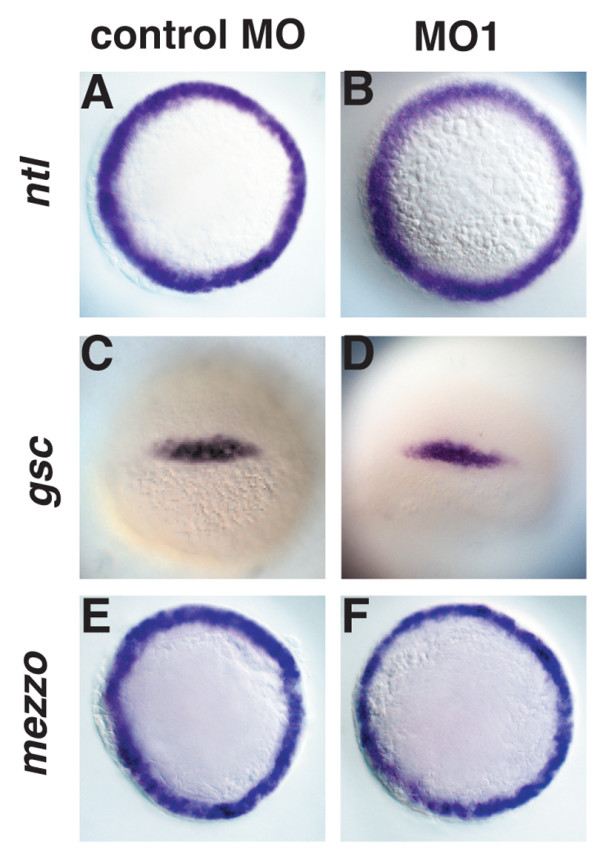
**Induction of mesoderm and endoderm are normal in *ogt *morphants**. Animal pole views of 5 hpf embryos (A, B, E, F) or dorsal views of 6 hpf embryos (C, D). *ntl *is expressed in the ring mesoderm and endodermal precursors around the margin in control MO (A) and MO1 (B) injected embryos. *gsc *(C, D) is expressed in dorsal marginal cells in *ogt *morphants (O) and controls (C). The *mezzo *transcription factor is expressed in a ring of endoderm precursors in control embryos (E). *mezzo *expression in the morphants is indistinguishable from controls (F).

The dramatic reduction in endoderm and the more subtle reduction in mesoderm and ectodermal derivatives could be explained by a failure to properly specify the germ layers before gastrulation. Therefore we examined expression of early mesoderm and endoderm markers in *ogt *morphants during the pregastrula stages. In controls, the *brachyury *homologue *no-tail *(*ntl*) is expressed in a marginal ring that includes all mesoderm and endoderm precursors at 5 hpf, as in wild type (N = 15) (Fig. 4A) [59]. This expression is not altered by injection of MO1 (Fig. 4B, N = 25) or MO2 (data not shown). Within the dorsal mesoderm, *flh *is expressed in the presumptive notochord, while *goosecoid *is expressed in the prechordal plate (Fig. 4C) [59,60]. Both *flh *(data not shown) and *gsc *(4D, N = 25) are expressed normally in the morphants. Finally, the *mezzo *transcription factor acts downstream of Nodal signals to specify endoderm, and is expressed in a ring at the margin (Fig. 4G) [61]. *Mezzo *expression in *ogt *morphants (Fig. 4F, N = 30) is indistinguishable from those injected with control MOs (Fig. 4E). Thus, the expression of early markers for mesoderm and endoderm appears normal when O-GlcNAc levels are reduced in *ogt *morphants. Therefore, we could find no evidence that O-GlcNAc modifications control the initial specification of the germ layers or establishment of the body axes. Instead, we conclude that morphological defects in *ogt *morphants observed at later stages are caused by the failure of precursor cells to survive or proliferate, or by aberrant morphogenesis.

### Variant 2 and Variant 4 Ogta isoforms are active *in vitro *and *in vivo*

**Figure 5:**
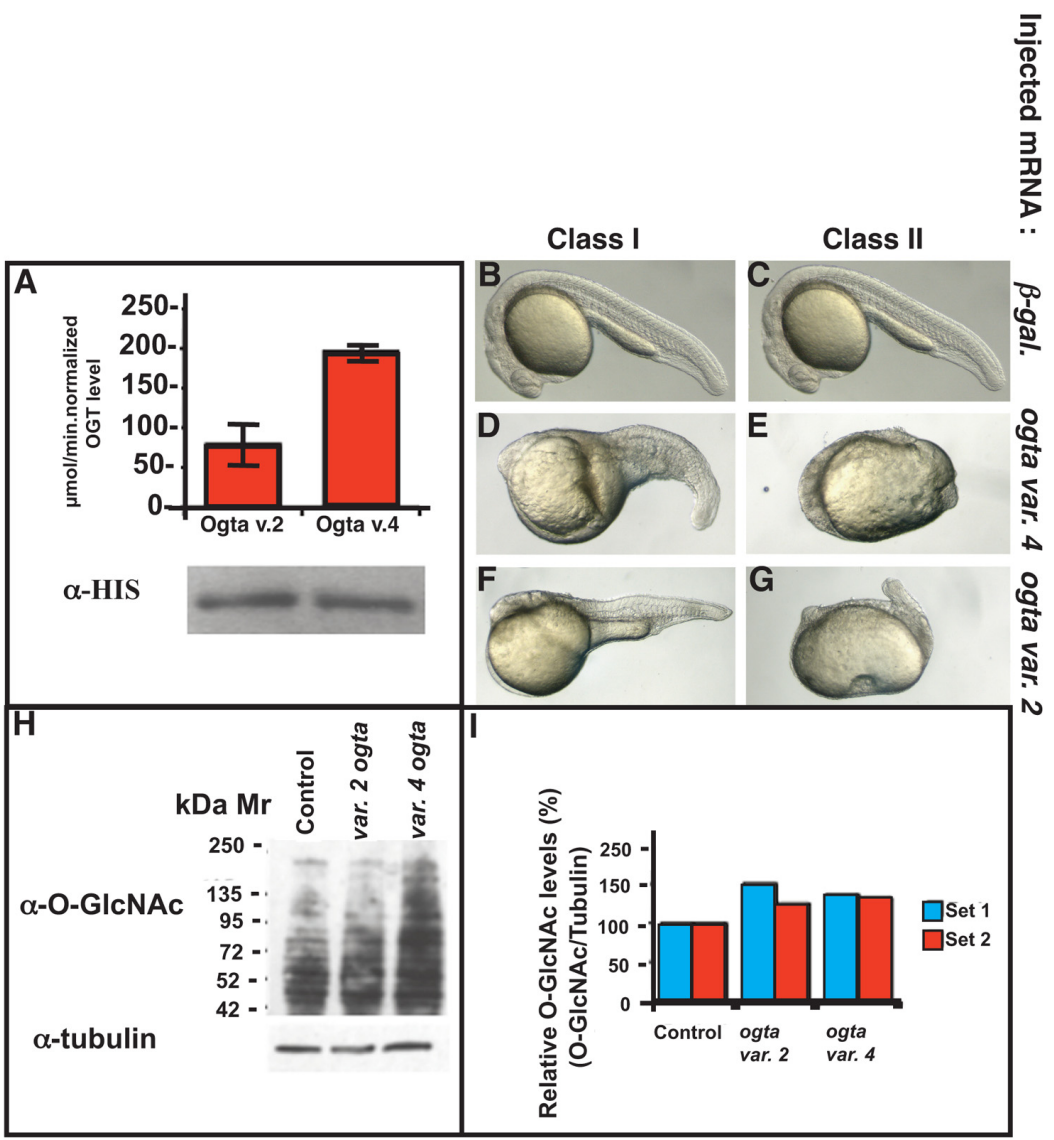
**Variant 2 and variant 4 Ogta proteins are active *in vitro *and *in vivo***. (A) *in vitro *Ogt activity assay of bacterially expressed, HIS-tagged variant 2 Ogta and variant 4 Ogta proteins. Both enzymes incorporate significant amounts of [3H]UDP-GlcNAc onto the synthetic CKII peptide identical to the human sequence, although variant 4 Ogta is more active than variant 2. A western blot of the bacterial lysates reveals that equivalent amounts of enzyme were added to the reactions. (B-G) Images of live 24 hpf embryos injected with 500 pg β-*galactosidase *mRNA (B, C), *variant 4 ogta *mRNA (D, E), or *variant 2 ogt *(F, G). Embryos injected with β-*galactosidase *mRNA developed normally (B, C). Overexpression of *variant 4 ogta *mRNA resulted in three phenotypic classes of embryos at this stage. Class I embryos had a visible, but shortened, body axis and reduced brain, including reduced or absent eyes (D). The body axis in Class II embryos was strongly reduced and in many cases, not discernable (E). Class III embryos are indistinguishable from wild type (not shown). Expression of *variant 2 ogta *mRNA produced the same range of phenotypes, including Class I (F) and Class II (G) embryos. (H, I) Western blot of protein extracts of 18 hpf embryos injected with 500 pg mRNA encoding *β-galactosidase *(lane 1), *variant 2 ogta *(lane 2), *variant 4 ogta *(lane 3). The blot was probed with the RL2 antibody, which recognizes the O-GlcNAc modification. This experiment was performed in biological duplicate (I) Quantification of the blot shown in (H, set 1) and a technical duplicate (set 2). B and C are duplicate images of the same control embryo.

Since tissues are reduced when Ogt is depleted, we asked if they are expanded when Ogt levels are increased. First, we asked if the variant 2 and variant 4 proteins encoded by our constructs displayed O-GlcNAc transferase activity in *in vitro *assays. We measured the ability of the bacterially expressed Ogt proteins to incorporate [^3^H]GlcNAc onto a synthetic CKII peptide, the best established *in vitro *acceptor substrate for Ogt [34]. A western blot of the bacterial lysates reveals that equivalent amounts of enzymes were added to the reactions (Fig. 5A). Both the variant 2 and variant 4 isoforms were active in this assay (Fig. 5A), although the specific activity of both zebrafish proteins is significantly lower than human Ogt in the same assay (data not shown). These results contrast to previous results indicating that variant 2 is inactive [46]. To test whether the isoforms were active in *vivo*, we injected embryos with 500 pg of mRNA encoding either β-galactosidase, variant 2 or variant 4 Ogta protein. We performed a Western blot on extracts from these embryos using the RL2 antibody, which is specific to the O-GlcNAc modification (see methods). In two separate experiments, O-GlcNAc levels were 30–50% higher in embryos overexpressing either variant 4 or variant 2 Ogta than in controls (Fig. 5H, I). We conclude that Ogta variants 2 and 4 are active both *in vitro *and *in vivo*.

To test the effect of increasing O-GlcNAc levels on embryonic development, we injected embryos with *ogta *mRNA. 100 pg or 250 pg *ogta *mRNA did not affect embryonic development (data not shown). By contrast, 500 pg mRNA encoding either variant 4 or variant 2 Ogta caused severe developmental abnormalities (Fig. 5). These defects are specific, since control embryos injected with 500 pg of β-*galactosidase *mRNA were indistinguishable from wild type (Figure 5B, C). Overexpression of variant 4 Ogta generated three classes of embryos at 24 hpf. Class I embryos had mildly reduced heads, eyes, notochords, and a shortened body axis similar to that observed in *ogt *morphants (Figure 5D, 23/129; compare with Fig. 2B, C). Class II embryos had more severe defects, including greatly reduced heads, missing eyes and no distinguishable body axis (Figure 5E; 20/129). Finally, class III embryos were indistinguishable from control embryos injected with β-*galactosidase *mRNA (data not shown; 35/129). The remaining embryos, comprising the largest group, died soon after gastrulation and therefore are not counted in the phenotypic classes at 24 hpf (51/129; see below). Overexpression of variant 2 Ogta produced similar defects at similar frequencies (Figure 5F, G). These results demonstrate that elevated O-GlcNAc levels disrupt embryonic development.

### Ogt overexpression disrupts the ectoderm, mesoderm and endoderm

**Figure 6:**
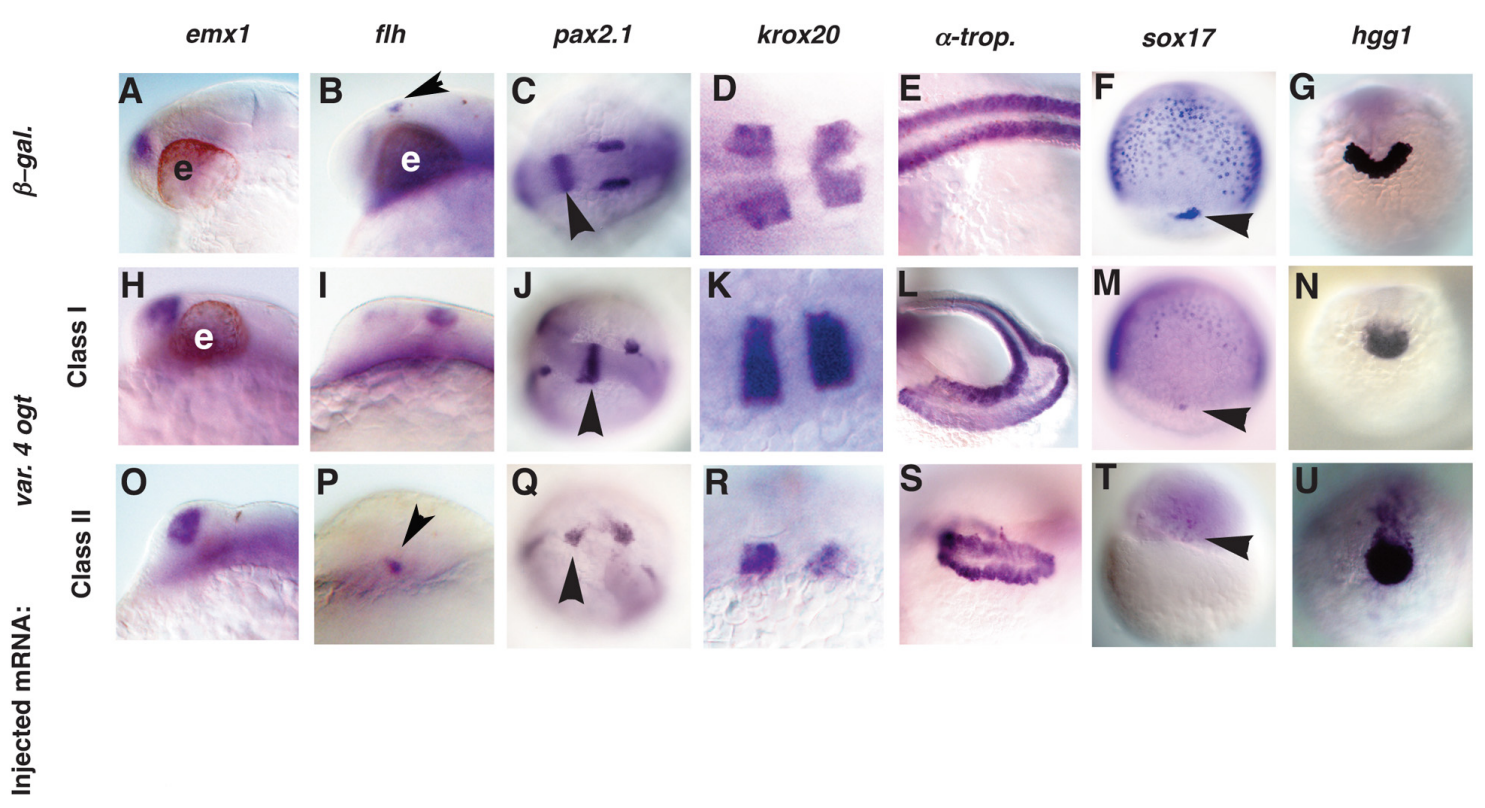
**Tissues from all three germ layers are reduced in Ogt overexpressing embryos**. Analysis of defects in Class I (H-N) and Class II (O-U) Ogt overexpressing embryos, compared to controls (A-G). Embryos at 24 hpf (A-E, H-L, O-S), 8 hpf (F, M, T) and 14 hpf (G, N, U) are shown. *emx1 *expression in class I (H) and class II (O) embryos is indistinguishable from controls (A). *flh *expression is slightly expanded in class I (I, arrowhead) and reduced in class II (P, arrowhead) embryos. In class I embryos, *pax2.1 *expression is normal in the MHB (J, arrowhead), whereas the otic vesicles are often disrupted (J). In class II embryos, *pax2.1 *expression is disrupted in the MHB (Q, arrowhead) and the otic vesicles are reduced or absent (Q). *krox20 *expression in rhombomeres 3 and 5 is indistinguishable in class I embryos (K) and controls (D). The rhombomeres are reduced and disrupted in class II embryos (R). α-*tropomyosin *expression is reduced and disorganized in class I embryos (L) and more severely reduced and disorganized in class II embryos (S). *sox17 *expression in endodermal precursors and dorsal forerunner cells is reduced in class I embryos (M) and severely reduced or absent in class II embryos (T) (arrowheads mark the forerunner cells). *hgg1 *is reduced in class I (N) and class II (U) embryos. Anterior to the left in A-E, H-L, O-S, dorsal views in F, M, T, and anterior views in G, N, U. e = eyes.

To determine which tissues are affected by increasing O-GlcNAc levels, we performed marker gene analysis on Ogt overexpressing embryos. In the experiments described below, we obtained similar effects for both Ogta variants. Unless otherwise indicated, we describe only those from embryos overexpressing Ogta variant 4. Expression of *emx1 *indicates that the dorsal telencephalon is present in Class I embryos (Fig. 6H, N = 20) and Class II embryos (Fig. 6O), and is roughly the same size as in β-*galactosidase galactosidase *mRNA injected embryos (Fig. 6A). *flh *is expressed in the epiphysis in Class I embryos (Fig. 6I; N = 20), as in controls (Fig. 6B, arrowhead). The epiphysis is greatly reduced or missing in Class II embryos (Fig. 6P, arrowhead), indicating that the size of the epiphysis varies widely in Ogta expressing embryos. In the brain, the transcription factor *pax2.1 *is expressed at the mid-brain/hind-brain (MHB) (Fig. 6C, arrowhead) boundary and in the more posterior otic vesicles [62]. In class I embryos, *pax2.1 *expression in the MHB boundary is not perturbed (Fig. 6J, arrowhead; 10/20), but the otic vesicles are often abnormally positioned. In class II embryos, *flh *expression at the MHB boundary is severely disrupted (Fig. 6Q, arrowhead; 5/20). In the hindbrain, *krox20 *stripes are normal, or slightly narrower in class I embryos (Fig. 6K; N = 25) as compared to controls (Fig. 6D). In class II embryos, the hindbrains are highly disorganized, and often display only a single stripe of *krox20 *expression (Fig. 6R). Thus tissues in the forebrain, mid-brain, and hindbrain are present, but are reduced and disorganized to varying extents in Ogt overexpressing embryos. Expression of the slow muscle marker, α-*tropomyosin *(Fig. 6E), is expressed in a reduced number of highly disorganized somites in Class I (Fig. 6L; 14/20) and Class II (Fig. 6S) embryos. In addition, both classes of embryos have fewer endoderm precursors at mid-gastrulation than controls, as revealed by *sox17 *expression (compare Fig. 6F and 6M). In class II embryos, only a few *sox17 *expressing cells are detected (Fig. 6T). *sox17 *expression in the dorsal forerunner cells is reduced or absent in both class I and II embryos (Fig. 6M, T, arrowheads). The hatching gland, a derivative of the prechordal plate, expresses the gene *hgg1 *(Fig. 6G) [63]. *hgg1 *expression is reduced in class I (Fig. 6N; 12/20) and class II (Fig. 6U) embryos. The reduced and disorganized endodermal, ectodermal and mesodermal tissue in Ogt overexpressing embryos strongly resembles the defects in embryos with reduced O-GlcNAc levels. Thus, increasing or decreasing O-GlcNAc levels results in similar defects. This indicates that O-GlcNAc levels must be tightly regulated within a narrow range for normal development to occur.

### Altering O-GlcNAc levels increases rates of cell death

**Figure 7:**
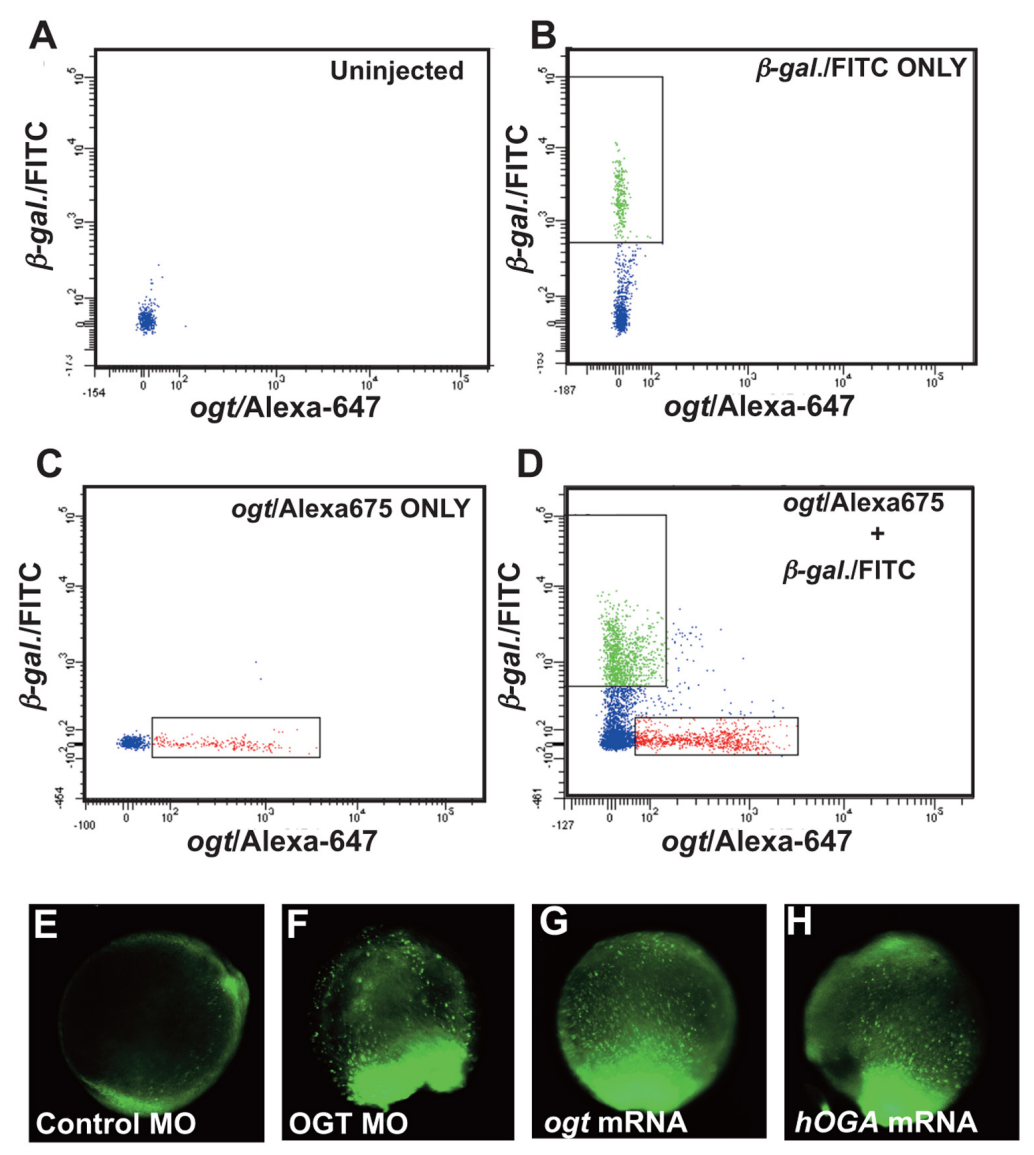
**Altering O-GlcNAc levels causes increased cell death**. Flow cytometry of dissociated cells from 18 hpf embryos. (A) Distribution of cells from uninjected embryos showing the position of non-fluorescent cells and debris. (B) Distribution of cells from embryos injected with β-*galactosidase *mRNA/FITC-dextran. The box indicates FITC positive cells between 5–10 μm in diameter. (C) Distribution of cells from embryos injected with *variant 4 ogta *mRNA/AlexaFluor-647-dextran. The box indicates AlexaFluor-647 positive cells between 5–10 μm in diameter. (D) Embryos injected with β-*galactosidase *mRNA/FITC-dextran and *variant 4 ogta *mRNA/AlexaFluor-647-dextran were mixed, dissociated, and fluorescent cells between 5–10 μm diameter were counted. In this experiment, we counted 67,711 FITC positive cells and 43,213 AlexaFluor-647 positive cells. (E-H) Images of live 10 hpf embryos stained with acridine orange, which labels dying cells. All embryos are oriented with the tailbud at the bottom of the panel. (E) In controls, only a few acridine orange positive cells are detected, indicating a low rate of cell death. By contrast, a large number of acridine orange positive cells are observed in *ogt *morphants (F), Ogt overexpressing embryos (G) and hOga overexpressing embryos (H), indicating a high rate of cell death.

We next asked if the smaller size of Ogta expressing embryos could be explained by a reduction in cell number. To test this, we used flow cytometry to count cells in embryos overexpressing Ogt and in controls (Fig. 7A–D). We injected embryos with a combination of either β-*galactosidase *mRNA and FITC-conjugated dextran, or *ogta variant 4 *mRNA and AlexaFluor-647-conjugated dextran. We mixed equal numbers of embryos from both groups at 18 hpf, when morphological differences between Ogt overexpressing embryos and the controls are apparent, and the embryos were homogenized to disperse the cells. In our analysis, we counted only cells in the 5–10 μm diameter range. In one experiment, we counted 67,711 cells in 20 control embryos and only 43,213 cells of the same size in 20 Ogt expressing embryos (Fig. 7D). Thus, control embryos contained 23% more cells than Ogt expressing embryos. Similar results were obtained in an independent experiment with fewer embryos (data not shown). This demonstrates that decreased cell number accounts for the reduced body size of Ogt overexpressing embryos.

The reduced cell number in Ogt overexpressing embryos could be explained by either an increase in apoptosis or a decrease in cell division. At 8 hpf, control embryos have only a few dying cells, as assessed by the extent of acridine orange staining (Fig. 7E). By contrast, injection of *variant 4 ogta *mRNA dramatically increases the number of dying cells, as shown by the increase in acridine orange fluorescence (Fig. 7G). Ogt overexpressing embryos have more dying cells than controls at every time point examined between 8 hpf and 24 hpf (data not shown). Thus, increased cell death could account for the decreased cell number in Ogt overexpressing embryos. *ogt *morphants and hOga expressing embryos display a similarly dramatic increase in the number of acridine orange staining cells (Fig. 7F, H). Thus, embryos with reduced levels of O-GlcNAc display elevated rates of cell death. The resulting decrease in cell number could explain the smaller size of *ogt *morphants and hOga expressing embryos. These results also confirm our earlier conclusion that the defects resulting from decreased levels of O-GlcNAc are similar to those observed in Ogt overexpressing embryos.

### OGT overexpression causes defective morphogenesis

**Figure 8:**
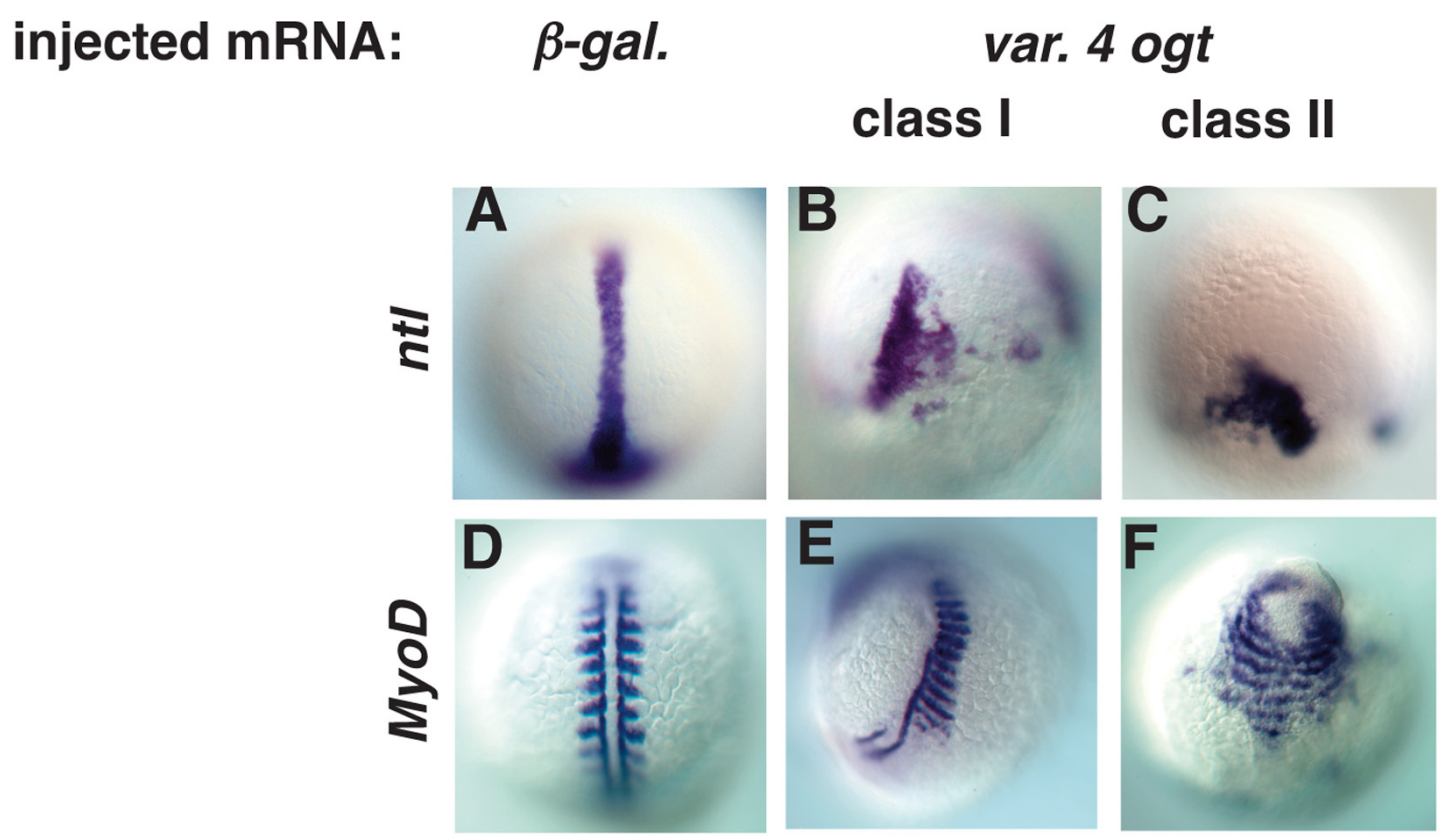
**Mesodermal derivatives are disorganized in Ogt expressing embryos**. Images of fixed embryos processed for *in situ *hybridization to reveal expression of *ntl *at 10 hpf (A-C) or MyoD at 14 hpf (D-F). At 10 hpf, *ntl *is expressed in the axial mesoderm in controls (A). *ntl *expression is truncated (B) in class I embryos, and more severely truncated in class II embryos (C). *MyoD *marks trunk somites on either side of the midline at 14 hpf (D). Some somites are missing in class I embryos, but many of the remaining somites are abnormally wide (E). In class II embryos, somites extend around the circumference of the embryos (F).

Although the increase in cell death could explain the reduced size of Ogt overexpressing embryos, it does not explain their aberrant morphology. To better understand when the embryos become disorganized, we examined markers of axial and paraxial mesoderm just after gastrulation, and during the segmentation stages. In control embryos after gastrulation, the cells of the axial mesoderm express *ntl *and extend along the midline (Fig. 8A). In Ogt expressing embryos, by contrast, *ntl *expression is truncated along the midline, resulting in a shortened body axis (Fig. 8B). In class II embryos, *ntl *expression is completely restricted to the margin (Fig. 8C). *MyoD *is expressed early in the segmented mesoderm that gives rise to the somites (Fig. 8D). Ogt expressing embryos have fewer somites than controls, with somites often forming in only half the embryo (Fig. 8E) [64]. In addition, the somites in class I embryos are often wider than those in controls (Fig. 8E). In class II embryos, the somites are extremely broad and nearly encircle the embryo (Fig. 8F). The aberrant morphology of the axial and paraxial mesoderm suggests that Ogt expression disrupts the morphogenetic movements associated with gastrulation.

### OGT overexpression causes delays in epiboly

**Figure 9:**
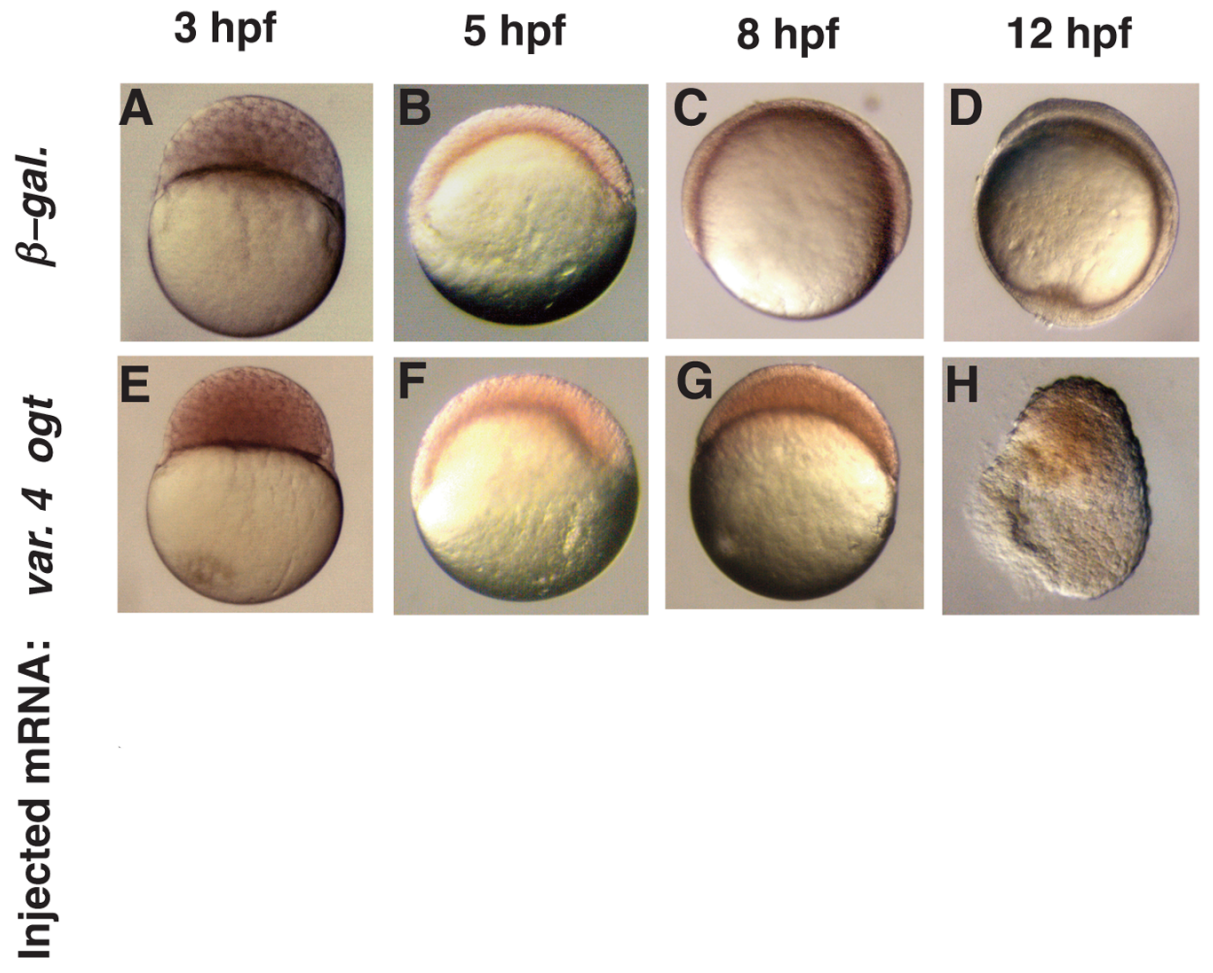
**Epiboly is delayed in Ogt expressing embryos**. A live time-course embryonic development of an embryo injected with *β-galactosidase *mRNA (A-D) or a sibling injected with encoding *variant 4 ogt *mRNA (E-H) at 3 hpf (A, E), 5 hpf (B, F), 8 hpf (C, G) and 12 hpf (D, H). Progression through the cleavage stages in *ogt *overexpressing embryos (E) is indistinguishable from controls (E). Epiboly initiates normally when Ogt is overexpressed, and these embryos reach 40% epiboly at 5 hpf (B), the same as controls (F). Ogt overexpressing embryos are stalled at 40% epiboly at 8 hpf (G), when controls are at 75% epiboly (G). Control embryos complete epiboly at 10 hpf and reach the 3 somite stage at 12 hpf (D). The most severely affected Ogt overexpressing embryos never complete epiboly and begin to degenerate at 12 hpf (H).

The first morphogenetic movement in the zebrafish embryo, called epiboly, begins before gastrulation when the blastoderm thins and spreads over the entire yolk [65]. As reported above (see Fig. 5), a large fraction of Ogt overexpressing cells die before 24 hpf, and many embryos examined before this stage had signs of defective morphogenesis (see Fig. 8). To determine if epiboly is disrupted by Ogt overexpression, we monitored the development of live embryos during a time course from 3–12 hpf. Ogt overexpressing embryos develop at the same rate as controls during the cleavage stages, and reach the 1000-cell stage at 3 hpf (Fig. 9A, E). Epiboly begins at 4.3 hpf in Ogt overexpressing embryos, as in wild type, and they reach 40% epiboly at 5 hpf (Fig. 9B, F). Occasional blastomeres separate from the blastoderms of Ogt overexpressing embryos during this period, and the remaining blastomeres are unevenly distributed around the margin (Fig. 9F). Gastrulation begins at 6 hpf, and control embryos reach mid-gastrulation two hours later (Fig. 9C). During the same time, epiboly does not progress in Ogt overexpressing embryos, which appear stalled at 40% epiboly (Fig. 9G). Less severely affected embryos progress through epiboly at a slower rate (data not shown). Thus, increased Ogt activity delays the late phase of epiboly, but does not affect the onset or initial progression of epiboly. At 12 hpf, when β-*galactosidase *expressing embryos are at the 3-somite stage (Fig. 9D), a large fraction of Ogt overexpressing embryos degenerate (Fig. 9H; 52/129). We observed similar defects along the same time course in embryos overexpressing *variant 2 ogt *mRNA and hOga (data not shown). We conclude that O-GlcNAc modifications control the activity of one or more proteins involved in the progression through epiboly.

### Ogt and hOga overexpression disrupts yolk microtubules and actin filaments

**Figure 10:**
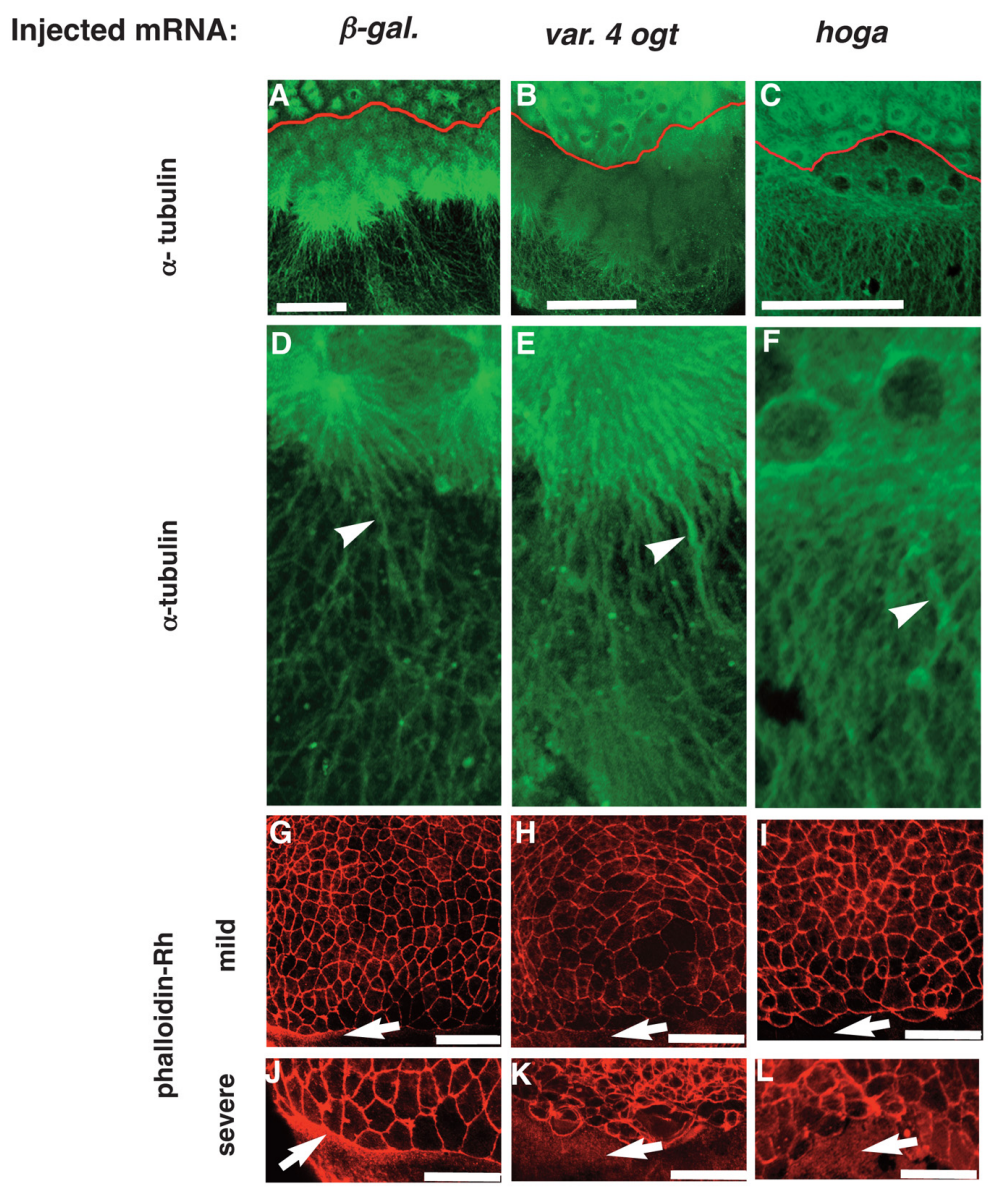
**Ogt overexpressing embryos have defects in the yolk cytoskeleton**. Confocal z-stacks of immunofluorescent images of embryos injected with β-*galactosidase *mRNA (A, D, G, J), *variant 4 ogt *mRNA (B, E, H, K) and *hOga *mRNA (C, F, I, L). Embryos at 4 hpf were incubated with the 12G10 1° antibody to reveal microtubule filaments (A-F) and 8 hpf embryos were incubated with rhodamine phalloidin (G-L). 12G10 reactivity reveals mitotic spindles in the blastomeres of controls embryos (A) Ogt expressing embryos (B) and hOga embryos (C). Within the yolk, long MT arrays extend around through the cortical cytoplasm toward the vegetal pole (A, D, arrowhead). In Ogt expressing embryos, many MTs appear thicker than normal and the arrays do not extend far towards the vegetal pole (B, E, arrowhead). Similarly, the MTs in the yolk are thicker than normal in hOga expressing embryos (F, arrowhead). Staining with rhodamine-phalloidin reveals actin filaments associated with the plasma membranes of EVL cells and the contractile actin ring in the yolk syncytial layer (YSL) (G-L). The contractile actin ring is apparent within the YSL of controls (G, J arrow), but not in OGT overexpressing embryos (H, K, arrow) or hOga expressing embryos (I, L, arrow). The EVL cells are highly irregular in size and shape in Ogt overexpressing embryos (H, K) and hOga expressing embryos (I, L). Red lines indicate the position of the YSL (A-C). Animal pole is to the top. Bars = 100 μm.

Disruption of the microtubule (MT) or actin-based cytoskeletal network in the yolk disrupts epiboly in a similar manner to that observed in Ogt and hOga overexpressing embryos [66-68]. Therefore we asked if the yolk cytoskeleton is disrupted when the enzymes are overexpressed. To examine the MT network, we utilized the antibody against α-tubulin (12G10) [69]. In 4 hpf controls, Z-projections of confocal images reveal a short latticework of MTs connecting the nuclei within the YSL and a network of longer MTs extending through the cortical yolk cytoplasm toward the vegetal pole (Fig. 10A, D), as previously described [67]. When Ogt activity is elevated, the MTs are shortened and they do not interconnect within the YSL (Fig. 10B, E). In addition, the MT filaments appear thicker than normal (Fig. 8D, E arrowheads). These results demonstrate that elevated Ogt activity disrupts the MT arrays in the yolk, either directly or indirectly. Similarly, the MT filaments appear shorter and thicker in embryos overexpressing hOga (Fig. 10C, F, arrowhead). This indicates that increasing or decreasing O-GlcNAc levels disrupts that yolk MT network.

In embryonic cells, F-actin associates with the cytoplasmic domain of E-cadherin in the cortical cytoplasm and decorates the cell membranes as visualized by rhodamine-phalloidin [70]. In control embryos, EVL cells are of roughly equal size and shape (Fig. 10G, J). By contrast, the shape and size of EVL cells are greatly disrupted by overexpression of Ogt (Fig. 10H, K) and of Oga (Fig. 10I, L). In controls, a punctate band of F-actin is detected within the YSL, underneath the EVL cells (Fig. 10G, J arrows)[68]. This band contracts during gastrulation and provides some force driving epiboly [68]. The band of actin in the YSL was missing in class I and class II Ogt overexpressing embryos (Fig. 10H, K, arrows). Similarly, the contractile actin ring was not detected in hOga overexpressing embryos (Fig. 10I, L). Thus, overexpressing hOga or Ogt results in a severe disruption of the actin and MT yolk cytoskeleton networks that drive epiboly movements.

### Ogt modifies human POU5F1 protein

**Figure 11:**
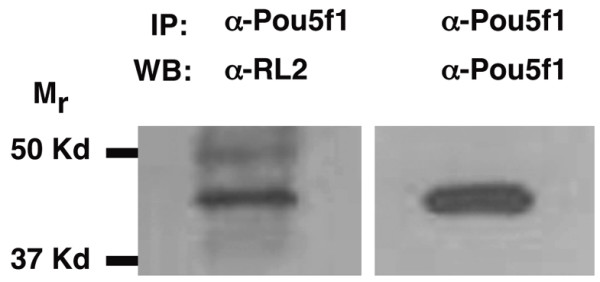
**Pou5f1 is modified by O-GlcNAc in human embryonic stem cells**. Western blot of protein immunoprecipitated by an anti-Pou5f1 antibody. The RL2 antibody recognizes a 43 KDa, O-GlcNAc modified protein that is immunoprecipitated from the nucleocytosolic fraction of human ES cells (line BG02) by an anti-Pou5f1 antibody. The immunoprecipitation was specific, since the nucleocytosolic fraction was precleared with a mixture of IgG and A/G PLUS agarose prior to incubation with the anti-Pou5f1 antibody. The anti-Pou5f1 antibody recognizes the same band when the blot is stripped and reprobed, confirming that Pou5f1 protein is modified by O-GlcNAc.

Some defects caused by increased levels of Ogt resemble those described in embryos lacking the maternal and zygotic function of the pou class transcription factor Spiel Ohne Grenzen/Oct4/Pou5f1 (MZSpg) [71,72]. The late phase of epiboly is delayed *MZspg *embryos, and cells in the EVL are irregularly shaped [71]. Secondly, *MZspg* embryos can display fused somites and truncated notochords, similar to those in embryos overexpressing Ogt (Fig. 8F) [72]. Finally, endoderm is reduced or absent in *MZspg *embryos, just as in embryos with increased or decreased O-GlcNAc levels (Fig. 2T, U; Fig. 6M, T)[73]. This raises the possibility that O-GlcNAc modifies Spg/Pou5f1 protein and regulates its function. Therefore, we asked if this protein is modified by O-GlcNAc. Since there is no antibody for zebrafish Spg/Pou5f1, we asked if human Pou5f1 is modified with O-GlcNAc in human embryonic stem cells (hESCs). We found that the O-GlcNAc specific monoclonal antibody RL-2 recognizes a 43 KDa protein immunoprecipitated by the Pou5f1 antibody (Fig. 11, left lane). This size is consistent with human Pou5f1 protein. Consistent with this, the same band reacts with the Pou5f1 polyclonal antibody when the blot is stripped and reprobed (Fig. 11, right lane). This demonstrates that Pou5f1 is modified by O-GlcNAc in hESCs. Given the high degree of homology between the human and zebrafish orthologues, it is likely that the zebrafish protein is also modified by O-GlcNAc [74,75]. It remains to be determined whether this modification regulates Spg/Pou5f1 activity.

## Discussion

In this work we used gain and loss of function experiments for the first time to study the role of Ogt and Oga during vertebrate development. We demonstrated that O-GlcNAc modifications control cell survival and epiboly movements in zebrafish embryos, but could find no evidence that they control early cell fate decisions. Furthermore, we observed the same range of defects whether Ogt protein was overexpressed or depleted. Since our enzymes are active in *in vitro *assays, this indicates that embryonic cells are highly sensitive to increases and decreases in the level of O-GlcNAc modifications. Finally, we identified Spg/Pou5f1 as a target for Ogt activity. These findings significantly extend previous genetic analyses of *ogt *function in embryos, and demonstrate that O-GlcNAc modifications regulate the activity of proteins involved in controlling morphogenetic movements.

### Zebrafish have two *ogt *paralogues

Unlike other vertebrates, zebrafish have two *ogt *genes, which we call *ogta *and *ogtb*, which arose during a recent gene duplication (Fig. 1A, B). Previous studies showed that transcripts from both loci encode O-GlcNAc transferase proteins, although one isoform (variant 2) failed to modify full-length p62 protein *in vitro *[46]. Two lines of evidence indicate variant 2 Ogta can modify proteins other than p62 (Fig 5). First, this protein catalyzes the addition of O-GlcNAc to the CKII peptide *in vitro *(Fig. 5A). This demonstrates that variant 2 Ogta is catalytically active, but does not provide any information about the endogenous substrates of this enzyme. Secondly, overexpression of variant 2 in embryos causes similar defects as overexpression of variant 4 Ogt, which would not be expected if the protein were catalytically inactive (Fig. 5F, G). In support of this conclusion, we found that variant 2 expressing embryos had increased O-GlcNAc levels as compared to control embryos injected with β-*galactosidase *mRNA (Fig. 5). We conclude that variant 2 Ogta is catalytically active. The apparent discrepancy between our results and those of Sohn and Do (2005) could be explained if variant 2 Ogta is unable to recognize p62, but can still recognize other substrates.

To reduce O-GlcNAc levels, we designed translation-blocking MOs against *ogta *and *ogtb*. Several lines of evidence indicate that the MOs specifically reduced *ogt *function. First, the *ogt *MOs prevented translation of mRNA encoding an Ogt-gfp fusion protein, whereas control MOs had no effect (Fig. 2E, F). Second, *ogt *MOs reduced the total amount of O-GlcNAc modifications in the embryo (Fig. 2V, W). This confirms that *ogta *and *ogtb *encode proteins that are required for O-GlcNAc transferase activity *in vivo*. Third, the defects in *ogt *morphants were rescued by co injection of a low dose of *ogt *mRNA (Fig. 3). Finally, overexpression of hOga, which removes O-GlcNAc from target proteins, caused similar defects to those observed in *ogt *morphants (Fig. 2D). The fact that MOs did not completely eliminate O-GlcNAc modifications can be explained by the persistence of Ogt protein translated from maternal transcripts prior to MO injection. In addition, each MO was designed to target only one *ogt *gene. Thus, Ogt protein continues to be synthesized from the non-targeted paralogue in *ogt *morphants.

### Gain and loss of *ogt *function produce similar defects

*ogt *morphants were smaller than controls and displayed a dramatic increase in apoptosis (Fig. 2B, C; Fig. 7). In addition, the morphants were deficient in endoderm and some mesoderm and ectoderm derivatives (Fig. 2). It is remarkable that we observe such strong phenotypes with *ogt *MOs (Fig. 2B, C) that reduced total O-GlcNAc modifications by only 20–35% (Fig. 2V, W). This raises the possibility that some Ogt targets may be more sensitive to reductions in O-GlcNAc than others. Furthermore, it suggests that additional roles for O-GlcNAc modifications could be uncovered if the amount of O-GlcNAc modifications were reduced to a greater extent, perhaps by co-injection of both MO1 and MO2. Given the dramatic increase in apoptosis in *Ogt *morphants (Fig. 7), however, it is possible that further reductions in O-GlcNAc levels would merely result in more dead or dying cells.

In a complementary approach, we overexpressed two variants of zebrafish Ogt. The resulting embryos were smaller than controls, and lacked endoderm and some ectoderm and mesoderm derivatives (Fig. 2). Cell counts revealed that Ogt overexpressing embryos had significantly fewer cells than controls, due to a concomitant increase in cell death (Fig. 7). Thus, embryonic development is severely disrupted by reducing O-GlcNAc levels by 20–35% as well as by increasing Ogt levels. This suggests that O-GlcNAc modifications must be maintained within a fairly narrow range for normal development. Surprisingly, decreasing O-GlcNAc levels produced the same range of defects as those observed in Ogt overexpressing embryos. This indicates that both the addition of O-GlcNAc to target proteins and its removal are essential for embryonic development. This is consistent with the idea that O-GlcNAc cycles on and off proteins, controlling a switch between two different active states [20].

### O-GlcNAc levels control the morphogenetic movements of epiboly

Our results indicate that gastrulation stage embryos are especially sensitive to changes in O-GlcNAc levels. First, we found that *ogt *expression decreases during gastrulation, consistent with the previous RT-PCR analysis (Fig. 1C–G) [46]. In *Xenopus*, Ogt protein levels also decrease during gastrulation [36]. This suggests that the downregulation of *ogt *during gastrulation has been conserved through evolution. In support of this conclusion, overexpression of Ogt or hOga causes a delay in epiboly (Fig. 9, 10 and data not shown). We did not observe delays in epiboly in *ogt *morphants, probably because these embryos continue expressing a significant amount of Ogt protein, as described above.

Control of morphogenetic movements represents a new role for O-GlcNAc modifications. To understand how O-GlcNAc might act during epiboly, we examined the MT and actin cytoskeleton in the yolk (Fig. 10). We found that the MT network in the cortical cytoplasm was disrupted in Ogt overexpressing embryos, with the MTs forming shortened, thickened filaments. The MT network is also disrupted in embryos overexpressing hOga. Similarly, the contractile actin ring in the YSL is absent in embryos overexpressing Ogt and hOga. We conclude that O-GlcNAc either directly or indirectly regulates that cytoskeletal network in the yolk.

The similarities between embryos overexpressing Ogt or hOga and those lacking the pou class transcription factor Spg/Pou5f1, suggest that Spg/Pou5f1 protein is a target of Ogt and that O-GlcNAc inactivates Spg/Pou5f1 protein. Consistent with this, we found that Pou5f1 protein immunoprecipitated from undifferentiated human ES cells is modified by O-GlcNAc. There is a high degree of sequence homology between human Pou5f1 and zebrafish Spg [74]. Mouse Pou5f1 can rescue *spg *mutants, indicating a high degree of functional conservation between the two proteins [75]. Therefore, it is highly likely that zebrafish Spg protein is also modified by Ogt. It is not known how O-GlcNAc modification regulates Pou5f1 function. Like many targets of Ogt, Pou5f1 is also modified by phosphorylation and the ability of Pou5f1 to stimulate transcription correlates with its phosphorylation status [76]. Depending upon which residues are modified, O-GlcNAc may stimulate or inhibit phosphorylation of Spg/Pou5f1 [20]. Further experiments are necessary to identify the residues modified by O-GlcNAc and to determine the functional consequences of O-GlcNAcylation on Spg/Pou5f1 protein.

## Conclusion

O-GlcNAc modifications are a poorly understood post-translational mechanism for regulating embryonic development. We demonstrated that embryonic O-GlcNAc levels control epiboly movements and cell survival, but do not regulate the initial cell fate decisions. Interestingly, gain and loss of *ogt *function produced the same range of defects. This indicates that the overall level of O-GlcNAc modification must be tightly controlled for normal embryonic development. Finally, O-GlcNAc modifies the pou class transcription factor Pou5f1 in human embryonic stem cells. Although the effect of this modification on Pou5f1 activity is not yet understood, it is possible that some of the defects described in this paper are mediated by improper regulation of Pou5f1.

## Methods

### Zebrafish strains, staging and imaging live embryos

We obtained wild type embryos by natural mating of WIK adults. Embryonic stages were determined by morphology and are reported as hours post fertilization (hpf) at 28.5°C, according to Kimmel et al., 1995. Live embryos were mounted in 3% methylcellulose in depression slides, oriented appropriately and photographed under a Leica MZFLIII stereo dissecting microscope using bright field optics. For the time course, embryos were mounted at 2.5 hpf and stored on slides in wet chambers at 28.5°C. Images were obtained at regular intervals.

### Isolating clones encoding variant 2 and variant 4 Ogta

We isolated a cDNA encoding the variant 2 isoform by PCR using a 15–19 hpf cDNA library as a template (gift from Bruce Appel, Vanderbilt University). Primers were designed from the full-length OGT sequence [46]. OGT full-length F: 5'-TTACGTCGACGAATGGCGAGCTCGGTG-3', OGT full-length R: 5'-ATGCGCGGCCGCGATCAGGTGCTCTCGC-3'. The PCR product was sequenced, and found to encode the variant 2 isoform. The cDNA was subcloned into the XbaI site of the pCS2 plasmid using the TOPO TA cloning kit (Invitrogen, Inc., Carlsbad, CA). To generate a cDNA encoding variant 4 OGT, we deleted exon 19 from variant 2 by long extension polymerase chain reaction (PCR) using primers flanking exon 19. OGTextF: 5'-TTTTTCTTAAGAAAAAGGCTGTTATTGACT-3'; OGTextR: 5'-AATTAGCTGGGTACCGGGCCCAATOGATTGGC-3'; exon19F: 5'-GCCACCACACAGATTAACAATAAA-3', exon19R: 5'-TTTTTCCCCCGCGGCCGCGAATTAAAAAACCTCCC-3'. To make the Ogt-GFP fusion protein using the Gateway system (Invitrogen, Inc., Carlsbad, CA), we amplified the N-terminal portion of Ogt using the following primers: BPogt1500F: 5'-GGGGACAAGTTTGTACAAAAAAGCAGGCTTAATGGCGAGCTCGGTGGGG-3'; BPogt1500R: 5'-GGGGACCACTTTGTACAAGAAAGCTGGGTAGCTGTGATGCGGATGCAC-3'. The resulting product was recombined into pDONR211. For the LR reaction, pDONRNogt was mixed with p3E-egfp and pCSDEST-2 and LR clonase following the manufacturers instructions.

### Phylogenetic analysis of *ogt *genes

ClustalW sequence alignment and phylogenetic analysis of *ogt *genes were performed on MacVector software. To construct the tree, we used the Neighbor joining method, and bootstrap values from 1000 replications are presented. We compared the *Danio rerio ogt *proteins (copy I: ENSDARP00000074945; copy II: ENSDARP00000054555), to those in *Arabidopsis thaliana *(SEC: NP187074; SPY: NP187761), *Mus musculus *(NP631883), *Homo sapiens *(AAH38180), *Takifugu rubripes *(ENSGACP00000024161), *Tetraodon nigroviridis *(GSTENP00017067001), *Gasterosteus aculeatus *(ENSTRUP00000024020), *Oryzias latipes *(ENSORLP00000007935), *Ciona intestinalis *(ENSCINP00000003035), *Caenorhabditis elegans *(NP001040861).

### Whole mount *in situ *hybridization

*in situ *hybridization was performed as previously described [77]. We used the following probes described in Table 1[78-81].

**Table 1:** Marker genes used in this study.

**Gene**	**Tissues**	**Reference**
*goosecoid *(*gsc*)	Prechordal plate (5 hpf)	[78]

*floating head *(*flh*)	Notochord (5 hpf)	[79]
	Epiphysis (24 hpf)	[54]

*no-tail *(*ntl*)	Pan-mesoderm (5 hpf)	[80]
	Notochord (10 hpf)	

*hgg1*	Hatching gland (12 hpf)	[63]

*α-tropomyosin*	slow muscle	[56]

*MyoD*	Developing somites	[64]

*mezzo*	Endoderm precursors	[61]

*sox17*	Committed endoderm and dorsal forerunner cells	[57]

*emx1*	Dorsal telencephalon	[53]

*krox20*	Rhombomeres 3 and 5	[55]

*pax2.1*	Midbrain-hindbrain boundary, otic vesicle	[81]

### Microinjection of mRNA and MOs

Sense mRNA was made using the Ambion mMessage mMachine RNA synthesis kit (Applied Biosystems, Austin TX). We injected 500 pg of *ogt *or *β-galactosidase *mRNA, or 250 pg *hOga *mRNA into chorionated embryos at the 1–4 cell stage. Embryos were allowed to develop and examined at 24 hpf or fixed at appropriate stages for *in situ *hybridization.

Translation blocking antisense morpholinos (MOs) to *ogt *transcripts were obtained from Gene Tools, Inc. (Philomath, OR). Their sequences are as follows: OGT MO1: 5'-CCACGTTCCCCACCGAGCTTGCCAT-3'; OGT MO2: 5'-TCTCCTTCACTCTTTACTGGATTCT-3'; OGT control (inverted): 5'-TCTTAGGTCATTTCTCACTTCCTCT-3'. To monitor efficiency of the injections, MO2 was tagged on the 3' with lissamine, and the inverted control was tagged at the 3' end with fluorescein. MOs were dissolved in distilled water at a concentration of 30 mg/ml and stored at -20°C. The working stock of each MO was diluted to 7.5 ng/nl in 0.2 M KCL prior to injection. Embryos were injected with 1 nl MO at the 1–4 cell stage. Embryos were allowed to develop and examined at 24 hpf or fixed at appropriate stages for *in situ *hybridization. For rescue experiments, embryos were injected first with 7.5 ng *ogt *MO, and subsequently injected with 10 or 50 pg *β-galactosidase *mRNA or *ogt *mRNA.

### Flow cytometry and cell death assays

We used flow cytometry to count cells in control and Ogt overexpressing embryos. We injected embryos with either a combination of *β-galactosidase *mRNA/FITC-conjugated dextran, or *ogt variant 4 *mRNA/AlexaFluor-647-conjugated dextran. Embryos were raised at 25°C until 18 hpf, when they were manually dechorionated. Prior to dissociation, we mixed an equal number normal embryos injected *β-galactosidase *mRNA/FITC-conjugated dextran with Class I and Class II embryos injected with *ogta variant 4 *mRNA/AlexaFluor-647-conjugated dextran. The embryos were placed inside a nylon mesh basket that had been inserted into a 5 mm glass dish containing 10 ml PBS and 1% BSA, and ground with a plastic pestle. Dissociated cells were collected in plastic tubes and placed at 4°C. To concentrate the cells, we pelleted the cells by centrifugation at 2000 g for 10 min. The pellet was resuspended in 250 μl PBS, 1% BSA. The mixture of cells was analyzed in a LSR-II Analyzer (BD Biosciences) with BD FACSDiva 4.2 software. To detect cells containing FITC-dextran, the samples were excited by a sapphire blue laser at 488 nm, and the emission wavelength was detected with a 530 +/- 30 band-pass filter. To detect cells containing AlexaFluor-647-dextran, samples were excited with a HeNe laser at 633 nm, and the emission fluorescence was detected with a 660 +/-20 band-pass filter. Size gating was fixed by running 5 and 10 μm diameter standard beads. Cells from a small number of uninjected control embryos served as a control for autofluorescence. Gates for the respective fluorophores were established by running samples with only FITC positive cells or only AlexaFluor-647 positive cells.

To assay for apoptosis, we dechorionated embryos on an agar-coated plate, and placed them in 5 μg/ml of acridine orange (Sigma Aldrich, St. Louis, MO) in embryo (E3) medium [82,83]. After 30 minutes, embryos were washed three times with embryo (E3) medium. The embryos were visualized using the FITC filter of a Leica MZFLIII fluorescent dissecting microscope (Leica Microsystems, Bannockburn, IL).

### Immunofluorescence and Confocal analysis of the cytoskeleton

At 8 hpf embryos were fixed with 4% paraformaldehyde in phosphate buffered saline (PBS) overnight at 4°C. Embryos were dechorionated and washed thoroughly with PBS containing 0.1% Tween (PBT). They were incubated in blocking solution containing 2% bovine serum albumin (BSA) at room temperature for 2 hours. To label F-Actin, embryos were incubated with rhodamine-phalloidin (Molecular Probes, Invitrogen, Inc., Carlsbad CA) at a dilution of 1:40 at room temperature in the dark. Embryos were washed 5 times in PBT, five minutes each wash. To make the stock solution, we dissolved rhodamine-phalloidin in methanol at a concentration of 6.6 μM and stored at -20°C. To analyze yolk MTs, embryos were manually dechorionated on agar-covered plates, and fixed at 4 hpf in Gard's Fixative for 4 hours at room temperature in small glass tubes [Gard's Fixative: 3.7% formaldehyde, 0.25% glutaraldehyde, 0.2% Triton X-100, 80 mM PIPES, pH 6.8, 5 mM EGTA, 1 mM MgCl2[84]]. They were washed once with PBS and stored overnight in 100% methanol at -20°C. The following day embryos were washed three times with PBS and incubated in 3.7 mg/ml NaBH_4 _for 6 hours at room temperature. The embryos were then washed 8 times in PBS and blocked for 1.5 hrs in blocking solution (1% DMSO, 0.5% Triton, 2% BSA, 2% sheep serum) and transferred to eppendorf tubes. Embryos were incubated in primary antibody (12G10 monoclonal anti α-tubulin antibody) at 1:100 dilution in block solution overnight. The following day, embryos were washed 3 times in PBS and incubated overnight in anti-mouse IgG-FITC (Zymed Laboratories) at a dilution of 1:200. Embryos were then washed with PBS and mounted and analyzed in a Leica TCS SP Confocal Microscope (Leica Microsystems, Bannockburn IL). Images were gathered in 10 μm sections and presented in Z-stacks.

### Bacterial expression of Ogt and enzymatic activity

Nucleotide sequences encoding variant 2 and variant 4 Ogt were cloned into pDONR (entry) and pDEST17 (expression) vectors via sequential recombination reactions according to manufacturer's instruction (Invitrogen, Carlsbad, CA). Expression plasmids containing the insert were transformed into *E. coli *BL21(DE3)pLysS competent cells (Stratagene, La Jolla, CA). To prepare bacterial lysate for activity assay, a colony of *E. coli *with each construct was culture in LB broth containing 100 μg/ml of ampicillin at room temperature at 250 rpm for 24 hours without induction. After harvesting, the cell pellets were processed as previously described [16]. *E. coli *strains containing human Oga and Ogt were also expressed in parallel for negative and positive controls respectively. To estimate the Ogt levels, *E. coli *lysates were resolved by precast 7.5% Tris-HCl precast minigel (Bio-Rad Laboratories, Hercules CA), and transferred to Immobilon-P transfer membrane (Millipore, Inc., Billerica, MA). After blocking with 3% BSA in TBS containing 0.1% Tween 20, the membrane was probed with HisProbe-HRP (1:1000 dilution, Thermo Scientific, Waltham, MA) and the final detection of HRP activity was performed using SuperSignal West Femto chemiluminescent substrate (Thermo Scientific, Waltham, MA) and exposed to CL-XPosure film (Thermo Scientific, Waltham, MA). Quantification of Ogt levels was done using Gel DocXR imager (Bio Rad Laboratories, Hercules CA). For enzymatic assay, 40 μg of crude lysate was added into reaction mixture containing a synthetic CKII peptide and UDP- [^3^H]GlcNAc as previously described [10] and allowed to react at 37°C for 35 min. The product was then purified via C18 column chromatography and the radioactive incorporation was measured by scintillation counter. The net incorporation of radioactive GlcNAc was subtracted by the reading of the negative control and normalizing the levels of Ogt before plotting.

### Immunoprecipitation of POU5F1 from hES cells

To prepare the nucleocytosolic fraction for immunoprecipitation, a pellet of human ES cells (BG02) was resuspended in 4 volumes of hypotonic buffer (5 mM Tris-HCl, pH 7.5/Protease inhibitor cocktail, Roche, Indianapolis, IN) and transferred into a 2 ml homogenizer. After incubating on ice for 10 min, the cell suspension was subjected to dounce homogenization followed by another 5 min incubation on ice. One volume of hypertonic buffer (0.1 M Tris-HCl, pH7.5, 2 M NaCl, 5 mM EDTA, 5 mM DTT/Protease inihibitor cocktail) was then added to the lysate. The lysate was incubated on ice for 5 min followed by another round of dounce homogenization. The resulting lysate was transferred to a microcentrifuge tube containing the Oga inhibitor PUGNAc (final concentration 10 μM) and centrifuged at 18,000 g for 25 min at 4°C [85]. The supernatant was transferred to new microcentrifuge tube. Protein concentration was determined using Bradford assay (Bio-Rad Laboratories, Hercules CA).

To immunoprecipitate Pou5f1, 1 mg of nucleocytosolic fraction was supplemented with 1% NP-40 and 0.1% SDS, and precleared with a mixture of normal rabbit IgG AC and protein A/G PLUS agarose (Santa Cruz Biotechnology, Santa Cruz CA) at 4°C for 30 min. Following clarification, the precleared supernatant was incubated for 3 hours at 4°C in the presence of goat polyclonal antibody raised against an N-terminal Pou5f1 peptide (Santa Cruz Biotechnology, Santa Cruz CA) at a 1:200 dilution. 40 μl of protein A/G PLUS agarose slurry was then added into the IP sample and the mixture was incubated for another 2 hours at 4°C. After washing with 4 ml of IP wash buffer (10 mM Tris-HCl, pH7.5, 150 mM NaCl, 1% NP-40, 0.1% SDS), the IP complex was eluted by adding 50 μl of Laemmli sample buffer containing β-mercaptoethanol and boiled at for 3 min. The sample was resolved by a 10% Tris-HCl precast minigel (Bio-Rad Laboratories, Hercules CA), and transferred to Immobilon-P transfer membrane (Millipore, Inc., Billerica, MA). After blocking with 3% BSA in TBS containing 0.1% Tween 20, the membrane was probed with RL-2 (1:1000 dilution) (Alexis Biochemicals, San Diego, CA). The final detection of HRP activity conjugated to the secondary antibodies was performed using SuperSignal West Femto chemiluminescent substrate (Thermo Scientific, Waltham, MA) and exposed to CL-XPosure film (Thermo Scientific, Waltham, MA). After developing the image on the film, the blot was then stripped with 0.1 M glycine (pH 2.5) at room temperature for 1 hour, wash with ddH2O and reprobed for Pou5f1 (1:1000 dilution) as described above.

To prepare the nucleocytosolic fraction for immunoprecipitation, a pellet of human ES cells (BG02) was resuspended in 4 volumes of hypotonic buffer (5 mM Tris-HCl, pH 7.5/Protease inhibitor cocktail, Roche, Indianapolis, IN) and transferred into a 2 ml homogenizer. After incubating on ice for 10 min, the cell suspension was subjected to dounce homogenization followed by another 5 min incubation on ice. One volume of hypertonic buffer (0.1 M Tris-HCl, pH7.5, 2 M NaCl, 5 mM EDTA, 5 mM DTT/Protease inihibitor cocktail) was then added to the lysate. The lysate was incubated on ice for 5 min followed by another round of dounce homogenization. The resulting lysate was transferred to a microcentrifuge tube containing the Oga inhibitor PUGNAc (final concentration 10 μM) and centrifuged at 18,000 g for 25 min at 4°C [85]. The supernatant was transferred to new microcentrifuge tube. Protein concentration was determined using Bradford assay (Bio-Rad Laboratories, Hercules CA).

To immunoprecipitate Pou5f1, 1 mg of nucleocytosolic fraction was supplemented with 1% NP-40 and 0.1% SDS, and precleared with a mixture of normal rabbit IgG AC and protein A/G PLUS agarose (Santa Cruz Biotechnology, Santa Cruz CA) at 4°C for 30 min. Following clarification, the precleared supernatant was incubated for 3 hours at 4°C in the presence of goat polyclonal antibody raised against an N-terminal Pou5f1 peptide (Santa Cruz Biotechnology, Santa Cruz CA) at a 1:200 dilution. 40 μl of protein A/G PLUS agarose slurry was then added into the IP sample and the mixture was incubated for another 2 hours at 4°C. After washing with 4 ml of IP wash buffer (10 mM Tris-HCl, pH7.5, 150 mM NaCl, 1% NP-40, 0.1% SDS), the IP complex was eluted by adding 50 μl of Laemmli sample buffer containing β-mercaptoethanol and boiled at for 3 min. The sample was resolved by a 10% Tris-HCl precast minigel (Bio-Rad Laboratories, Hercules CA), and transferred to Immobilon-P transfer membrane (Millipore, Inc., Billerica, MA). After blocking with 3% BSA in TBS containing 0.1% Tween 20, the membrane was probed with RL-2 (1:1000 dilution) (Alexis Biochemicals, San Diego, CA). The final detection of HRP activity conjugated to the secondary antibodies was performed using SuperSignal West Femto chemiluminescent substrate (Thermo Scientific, Waltham, MA) and exposed to CL-XPosure film (Thermo Scientific, Waltham, MA). After developing the image on the film, the blot was then stripped with 0.1 M glycine (pH 2.5) at room temperature for 1 hour, wash with ddH_2_O and reprobed for Pou5f1 (1:1000 dilution) as described above.

## Authors' contributions

DMW participated in the design and performance of all experiments described in this paper, with the exceptions of those shown in Fig. 7, 3 and 10C, F, I, L. In addition, she wrote the first draft of the manuscript and aided in subsequent revisions. CFT designed and performed the *in vitro *glycosylation assays (Fig. 5), the RL2 Western Blot in Fig. 2, and the Pou5f1 IP experiment (Fig. 11). She wrote the corresponding sections in the Materials and Methods sections. YS designed and performed the flow cytometry experiments with the assistance of KDK, and performed the acridine orange stains (Fig. 7). YS performed the confocal analysis of hOga expressing embryos (Fig. 10C, F, I, L) with the assistance of DW. DW designed the protocol we used to visualize MTs in early zebrafish embryos, and assisted with the confocal analysis. SG made the *ogt-gfp *fusion construct and assisted with the injections to test the efficiency of ogt-MOs. LW provided expertise on O-GlcNAc and supervised the design, execution and interpretation of the biochemical experiments. STD conceived of the project and designed the experimental plan. He also supervised the design, execution and interpretation of all experiments in zebrafish embryos. After the first draft, STD revised the manuscript with the assistance of DMW, CFT, and LW. All authors have read and approved the final manuscript.
